# An overview of forensic drug testing methods and their suitability for harm reduction point-of-care services

**DOI:** 10.1186/s12954-017-0179-5

**Published:** 2017-07-31

**Authors:** Lane Harper, Jeff Powell, Em M. Pijl

**Affiliations:** 1 University of Lethbridge, 4401 University Drive, Lethbridge, AB T1K 3M4 Canada; 2 Carleton University, 1125 Colonel By Dr, Ottawa, ON K1S 5B6 Canada

**Keywords:** Harm reduction, Substance abuse, Street drugs, Drug overdose, Drug users, Drug effects, Drug-related side effects and adverse reactions, Drug evaluation

## Abstract

Given the current opioid crisis around the world, harm reduction agencies are seeking to help people who use drugs to do so more safely. Many harm reduction agencies are exploring techniques to test illicit drugs to identify and, where possible, quantify their constituents allowing their users to make informed decisions. While these technologies have been used for years in Europe (Nightlife Empowerment & Well-being Implementation Project, Drug Checking Service: Good Practice Standards; Trans European Drugs Information (TEDI) Workgroup, Factsheet on Drug Checking in Europe, 2011; European Monitoring Centre for Drugs and Drug Addiction, An Inventory of On-site Pill-Testing Interventions in the EU: Fact Files, 2001), they are only now starting to be utilized in this context in North America. The goal of this paper is to describe the most common methods for testing illicit substances and then, based on this broad, encompassing review, recommend the most appropriate methods for testing at point of care.

Based on our review, the best methods for point-of-care drug testing are handheld infrared spectroscopy, Raman spectroscopy, and ion mobility spectrometry; mass spectrometry is the current gold standard in forensic drug analysis. It would be prudent for agencies or clinics that can obtain the funding to contact the companies who produce these devices to discuss possible usage in a harm reduction setting. Lower tech options, such as spot/color tests and immunoassays, are limited in their use but affordable and easy to use.

## Background

Given the current opioid crisis in Canada [1–3] and around the world [4], harm reduction agencies are seeking to help people who use drugs to do so more safely. Harm reduction sites and/or clinics are increasing in number and service provision across the world, making it crucial to provide point-of-care workers with the tools and knowledge necessary to provide proper care for people who use drugs. Drug, pill, and substance testing are increasingly being used as a harm reduction strategy throughout the world [5–8] to decrease the risk of adverse effects. Indeed, various approaches to drug testing have been around, even in North America, for decades [9–11]. More recently, in Canada, drug testing is becoming more common at music festivals [12]. In Canada, the Standing Committee on Health [13] recommended that the Government of Canada grant exemptions under the Controlled Drugs and Substances Act so that drug testing could occur at designated sites. While there are certainly legal hurdles to overcome when it comes to drug testing [6], there are three primary advantages to testing drugs before they are consumed: short- and long-term adverse effects (including overdose and fatality) can be avoided by the person using the substance; other institutions (such as hospitals) and public health authorities can be made aware when a lethal or novel substance begins to circulate; and, a global picture of drugs in circulation can be generated [5, 14–16]. The goal of this paper is to describe the most common methods of testing chemical substances in both laboratory and point-of-care settings. We will conclude with recommendations for point-of-care testing of illicit substances. In this paper, we use the term “drug testing” to refer to the forensic testing of illicit substances in their intended consumption form. Please note that the legal issues surrounding, and the service models of, drug testing are beyond the scope of this paper.

## Introduction to substance testing methods

The following methods have been validated by the Scientific Working Group for the Analysis of Seized Drugs (SWGDRUG). Scientific Working Groups consist of scientific subject-matter experts who collaborate to determine best practices and develop consensus standards. As such, these methods have been proven to be effective in the analysis of unknown (forensic) examination of illicit substances and are therefore also the best methods to use in identifying unknown substances. Not all of these methods are easily accessible in a point-of-care framework, as some require high technical knowledge and/or a laboratory setting. Therefore, any of the following methods may be suitable on a case-by-case basis. This is due to the fact that some clinics may be able to easily access more discriminatory methods, through direct funding or industry partnership, whereas some clinics may have to rely on less precise testing methodologies and equipment due to lack of funding or support.

More discriminatory methods carry a much larger price tag to invest in the proper equipment. This may require community partnerships or a serious cost-benefit analysis or both. To keep the information precise and to attempt to interpret some of the associated technical details, the methods have been broken down into subheadings. Each method has three subheadings: “How does it work?” (a brief discussion of the theory behind the method), “What substances can be detected and how accurately?”, and “How easy is it to use?” The methods have also been broadly assigned into two larger categories: most discriminatory, or methods that will accurately identify a substance/mixture and that also have the potential to quantify the amount of substance, and least discriminatory, or methods that presumptively identify a substance and/or mixture without quantification. At the end of the paper, there will be a recommendation section that will focus strictly on the best methods/devices considering only point-of-care situations. The methods are summarized in Table 1.

**Table 1:** Summary of drug testing technologies and methods, and definition of terms

	Method	Discrimination	Substances	Identify (qualify)	Amount (quantify)	Destroy sample?	Lab	Point of care	Cost (USD)	Ease of use	Time required for results
Most discriminatory	Mass Spectrometry	★★★★★	Virtually any	✓	✓	Yes	✓		$5000 (used, older)–200.000+ (new, advanced models) Plus recurring costs (reagents, servicing)	Intermediate–advanced (depending on model)	Minutes
	Infrared spectrometry	★★★★★	Virtually any	✓	✓	No	✓	✓	$4000 (used, older)–100,000 (new, advanced models) Portable: 10,000–60,000 Plus recurring costs (licensing, servicing)	Basic–advanced (depending on model)	Second to minutes (Inc. Portable)
	Raman Spectroscopy	★★★★★	Virtually any	✓	✓	No	✓	✓	$5000 (used, older)–100,000 (new, advanced models) Portable: 10,000–60,000 Plus recurring costs (licensing, servicing)	Basic–advanced (depending on model)	Seconds to minutes (Inc. Portable)
	X-ray diffractometry	★★★★	Crystalline (solids)	✓	✓	No	✓		$50,000–250,000+ Plus recurring costs (reagents, servicing)	Advanced—expert	Minutes to hours
Least discriminatory	Thin-layer chromatography	★★★	Most common drugs of abuse; possibly not some novel psychoactive substances	✓		Yes	✓	✓	Initial supplies: $1000–3000 Recurring costs: $100–1000 per month depending on volume of usage (bulk reagents)	Basic—intermediate	Minutes to hours
	Ultraviolet spectroscopy	★★★	Most common drugs of abuse	✓		No	✓	✓	$3000–10,000	Basic—intermediate	Minutes
	Spot/color tests	★★	Most common drugs of abuse; must be already characterized (i.e., possibly not some)	✓		Yes	✓	✓	Approximately 2–5 dollars per test (in house) Recurring costs: $100–500 per month depending on volume of usage (bulk reagents)	Basic—intermediate	Seconds to minutes
	Microcrystalline tests	★★	Several	✓		Yes	✓	✓	Approximately 2–4 dollars per test (in house) Recurring costs: $100–500 per month depending on volume of usage (bulk reagents)	Intermediate—advanced	Minutes
	Immunoassay	★★	Various metabolized drugs in urine samples	✓		Yes	✓		$5000–20,000 for initial equipment (analyzer) Recurring costs: $300–1000 per month depending on volume of usage (bulk reagents)	Intermediate—advanced	Minutes
	Urine dipstick test	★★	Fentanyl	✓		Yes		✓	Approximately 1–5 dollars per test (in house) Recurring costs: $50–400 per month, depending on volume of usage	Basic—intermediate	Seconds to minutes

None—requires absolutely no knowledge or training. Basic—requires simple (hours to days) training by someone who knows the technique or theory, but is not an expert in the field, i.e., someone with intermediate, advanced, or expert skill/knowledge. Intermediate—requires a higher level of knowledge or skill, although that may be obtained from either following previous instructions obtained (i.e., gaining experience) while a basic user, or from further instruction from an advanced or expert level user. Usually requires days to weeks of experience depending on technique. Advanced—requires some college or university level theory or experience. Usually taught directly or indirectly by an expert in the subject/field. Occasionally, an intermediate user may become advanced without advanced education through diligence and interest. Requires weeks to months (a typical semester). Expert—an expert in the area, almost always has post-secondary education related to the field. May be a bachelor, master, or PhD holder or very high specialized training. Instruction may also be provided by someone from a device manufacturing company who provides a seminar or some sort of direct training in usage of a technique or device. Typically always requires months to years depending on difficulty of the subject

## Most discriminatory

### Mass spectrometry

#### How does it work?

Mass spectrometry (MS) is the most discriminatory of the drug testing techniques. Mass spectrometry measures the precise molecular mass of ions as determined by their mass to charge ratio (*m*/*z*) and is the current gold standard in forensic drug analysis [17]. In general, mass spectrometry requires separation, ionization, and finally detection. Separation can be accomplished through gas chromatography (GC), liquid chromatography (LC), or capillary electrophoresis (CE). There are various ionization methods. The most commonly used in analysis of illicit substances are electron ionization (EI), atmospheric pressure chemical ionization (APCI), electrospray ionization (ESI), matrix-assisted laser desorption ionization (MALDI), atmospheric pressure photoionization (APPI), fast atom bombardment (FAB), and more recently direct analysis in real time (DART). Ionization methods can be grouped into hard or soft techniques.

Hard techniques like EI, FAB, and APCI cause molecules to fragment generating complex mass spectra. Fragmentation is useful in analysis because molecules have known fragmentation patterns. A spectral database allows for a computer to quickly match spectra and determine the molecular species. Hard techniques are limited to detecting small molecules. Most illicit drugs are small molecules with the exception of drugs of a biological nature being consumed in their raw form.

Soft ionization techniques such as MALDI and ESI minimize fragmentation and allow for the molecules being analyzed to remain intact. Soft ionization techniques are useful for large biomolecules such as proteins.

DART is of particular interest as it allows non-destructive testing, is fast, and can quickly quantify when used with an internal standard. A pill can be held in front of the gas stream and within seconds determine the molecular species present. DART does not require separation of each molecular species prior to analysis allowing untrained personnel to collect data [18].

#### What substances can be detected and how accurately?

Virtually, any substance can be identified using MS in combination with a separation (chromatographic) technique. Sensitivity of current mass spectrometers allows for detection of analytes at concentration in the attomolar range (10^−18^) [19]. MS has increased sensitivity over some other analytical techniques as the analyzer, a mass-charge filter, reduces background interference (i.e., a clearer reading/analyte fingerprint can be produced). It demonstrates excellent specificity due to characteristic fragmentation patterns, high resolution, and unique filtering abilities available especially in tandem or higher order mass spectrometry [20].

MS provides information about molecular mass and isotopic abundance of elements and temporally resolved chemical data, allowing for highly accurate identification. Newer devices are easier to utilize and much smaller than older versions. Interfacing with computers allows for refined database searches, making the drug identification process easier.

A major drawback of MS is that the tested sample taken from the supply is destroyed by the testing process (DART being an exception). Only a very small sample size (milligrams) is required. There are also continuing costs due to consumable materials required, and some of these consumables are poisonous/hazardous. Complex mixtures must be separated with a chromatographic technique (either gas or liquid chromatography) to correctly identify each constituent (unless using DART).

#### How easy is it to use?

The expertise required to utilize this technology is intermediate to expert (for definitions of terms in context with this paper please, see Table 1). Individuals should have some theoretical knowledge of how the technology and specific instrument work and specialized training from an expert. The cost of a mass spectrometer can vary from US$5000 to US$1,000,000. While an older used mass spectrometer may be less expensive upfront, it is not necessarily suitable for point-of-care drug testing. There are also considerable ongoing operational costs, such as chromatography (separation) reagents, gas consumables (nitrogen, helium, etc.), sample preparation items, and routine maintenance and service. Some labs offer MS services with costs between US$5 and US$100 per sample.

### Ion mobility spectrometry

#### How does it work?

Ion mobility spectrometry (IMS) separates and identifies ions based on their speed through a carrier gas. Ion mobility is dependent on three molecular characteristics: the charge, reduced mass, and the collision cross section of the ion. IMS requires ionization before samples are passed into the instrument. This can be accomplished by ESI, MALDI, APPI, and coronal discharge or by using radioactive sources such as nickel-63.

There are many designs for ion mobility spectrometers including drift tube, ion trap, traveling wave, high-field asymmetric waveform, and differential mobility types. Drift tube IMS determines the ion mobility based on the amount of time it takes for ions to reach the detector. Many modern instruments use a drift tube for analysis.

Of interest is the field asymmetric subtype of the high-field asymmetric waveform IMS. A field asymmetric ion mobility spectrometer (FAIMS) uses a high (strong) electric field to control the movement of the ions through a physical filter. A pulsing electric field can then be applied to select for ions with specific ion mobility. Only ions with the specifically selected mobility will be able to maintain a stable trajectory through the filter. The others will crash into the side walls and not reach the detector.

#### What substances can be detected and how accurately?

Any small molecule of illicit substance can be detected very quickly and accurately. FAIMS sensitivity is based on multiple characteristics of both the ion of interest and the physical environment. IMS can detect one molecule in a billion (ppb) and is very selective. IMS selectivity can be further enhanced when using FAIMS. FAIMS is able to operate in environments with high levels of interference with minimal adjustment to operating conditions [21]. IMS is non-destructive and only requires a very small sample if a quantitative method calls for destructive testing. Determination is very quick and can be accomplished in a few seconds even for a complex sample.

#### How easy is it to use?

IMS instruments do not require a trained operator. They can be used to quickly analyze a sample. Identification does require a database of known molecules to compare the sample against. The process of building a database would require a trained chemist using another technique or a standard. Once built, a database could be referenced from any instrument without additional technical help [22]. Quantification is possible when using internal standards or prebuilt methods. IMS is regularly used by law enforcement agencies at airports to detect narcotics and explosives. Minimal maintenance, ease of use by non-technical personnel, low cost, fast and accurate determination, minimal cost of consumables, and robust methodologies make IMS one the best choices for drug identification.

### Infrared spectrometry

#### How does it work?

Infrared (IR) spectroscopy is another highly discriminatory method and is based on the measurement of the amount of IR radiation which is absorbed or emitted by a sample as a function of wavelength. A spectrum is obtained by passing infrared radiation through a sample and determining the amount of the incident radiation (radiation that actually hits the molecule rather than passing through) that is absorbed at each IR frequency [23]. Interpretation of the spectra allows for determination of molecular functional groups. The IR spectra of a pure molecular compound provides a distinctive fingerprint which can be easily differentiated from the IR absorption pattern of other compounds, including compounds with the same chemical formula, but a different arrangement of atoms in the molecule (known as isomers) [23]. An advantage of IR techniques is that virtually, all compounds have IR active vibrational modes and can therefore be investigated both qualitatively and quantitatively. However, quantitative analysis can pose a problem with unknown samples and mixtures. The spectroscopic expertise required to forensically analyze and quantify a substance may be difficult or impossible to find in harm reduction clinics. Most papers that describe relatively simple quantification methods are carried out in pharmaceutical research with controlled standards, methodologies, and standards. While quantification of unknown substances is technically possible, it really comes down to a case-by-case basis and is generally a laborious process undertaken by advanced to expert level technicians and chemists in forensic laboratories. It is highly unlikely that quantification would be viable using this technology in this kind of setting. Recent advances in IR technology have allowed for the development of portable IR devices.

#### What substances can be detected and how accurately?

When reference spectra are available, most compounds can be unambiguously identified based on their IR spectra. Drugs can be identified through a searchable database (such as http://webbook.nist.gov/). IR cannot distinguish enantiomers (similar to MS) [24]. According to the SWGDRUG [24], IR can produce structural information that will provide sufficient selectivity that generates the highest discriminating capability. IR can discriminate between diastereomers (such as pseudoephedrine and ephedrine) and free base/acid and salt forms. Free base/acid and salt forms refer to differences in physical properties that can alter the application of the substance. Free base is usually more volatile and normally has a lower boiling point, allowing the substance to be smoked. The salt form is usually more stable and tends to be crystalline and dissolvable in water, allowing for ingestion, insufflation (inhaling through the nose), or injection. A common example is crack cocaine (free base) and cocaine (salt); they are in fact the same drug (cocaine), and the actual effect on the body is the same, but due to different absorption and dosages based on method of use, it is possible to observe a spectrum of differing responses to each of the drugs. One of the notable benefits of IR spectroscopy is that it does not destroy the sample provided—an important consideration when working with drugs and the people who use them. As well, it requires only a very small sample size in the range of milligrams or less. Additionally, samples can be studied in virtually any physical state (primarily solid or liquid). Interference is very common and causes difficulty in identification.

#### How easy is it to use?

The level of expertise required to use this technology varies depending on the device. There are portable IR devices on the market that have been optimized for basic to intermediate knowledge base, such as by outreach workers. These devices can analyze the obtained spectrum and search internal databases to display the identified substance or substances in a mixture (to a certain concentration, based on the specifications of a given device). This is considered presumptive or qualitative testing, in that it may only give an accurate breakdown of the constituents of a substance or mixture and sometimes offer a semi-quantitative analysis (i.e., rank-ordered most to least in a mixture). For quantification (as percent mass by total mixture weight), Sorak et al. have shown that some portable IR devices may be used for low error quantitative analysis [25]; although in order to interpret the obtained spectrum in the these devices in a quantitative manner, advanced to expert level knowledge is required as the devices do not perform this task for the user. Many other IR devices also require at least an intermediate level understanding of the procedures and some require advanced to expert knowledge to correctly analyze and quantify the substances (including operation of the equipment and database searching). Costs of IR devices can be anywhere from the low thousands to US$60,000 and above.

### Raman spectroscopy

#### How does it work?

Raman spectroscopy is an optical technique based on the inelastic scattering of radiation after it interacts with matter. The interaction of incident radiation with the molecules of the substance gives spectral vibrational information [26]. The technique involves shining a laser on a sample and detecting the scattered light. A small amount of the scattered light is shifted in energy from the laser frequency due to electromagnetic and molecular interactions in the sample [26]. Plotting the intensity of the shifted light versus frequency gives a Raman spectrum of the sample. An exciting breakthrough in this technology is the development of handheld, portable Raman spectrometers. Many of these devices, most notably the TruNarc device by Thermo Fisher Scientific, have been optimized for drugs of abuse detection with simple “point and shoot” action. These devices also search databases in real time at a device level and give a clear readout of what substance(s) were detected.

#### What substances can be detected and how accurately?

Virtually, any drug can be identified with Raman spectroscopy. It can be used to determine active pharmaceutical ingredients (APIs) as well as molecules with the same chemical formula but different molecular arrangement and polymorphs. This is important as many of the novel psychoactive substances that have been emerging are isomers, derivatives, and analogues of many of the classical drugs of abuse. Being able to differentiate between small differences in physical or chemical structure aids greatly in unambiguous identification. Portable Raman spectroscopy has even been reported to be able to detect the date-rape drug rohypnol (flunitrazepam) in spiked beverages [27].

Raman spectroscopy may have difficulty in identifying substances that exhibit strong fluorescence. These substances tend to be plant-based narcotics such as heroin. However, with proper sample preparation, it is possible to analyze even these substances. The TruNarc Raman spectroscopy device has been shown to have a very high level of agreement with laboratory results (MS) for cocaine, heroin, and methamphetamine; inconclusive results are generally related to illicit substances that are present at extremely low percentages of the total mixture. Some studies have indicated that cocaine can be detected at concentrations as low as 5% when the cocaine was cut with sorbitol [28]. Others have detected amphetamine residues (milli- to micrograms) on paper currency using Raman spectroscopy [29]. It must be stressed that the particular technology discussed (TruNarc by Thermo Fisher Scientific) does not offer quantitative data in its “point and shoot” identification action, although it does offer highly accurate and extremely easy-to-use qualitative testing. The Raman technique as a whole is able to identify and quantify (depending on the device) a wide range of illicit drugs, even in the presence of contaminants and adulterants [26]. Given that there are many substances used to “cut” illicit drugs, this feature is an important one.

RS is rapid and non-destructive, does not require chemical reagents, can detect separate substances in mixtures, is not subject to interference from water or moisture, and importantly, can detect substances through transparent packaging (such as plastic bags and glass containers). Little or no sample preparation is required, although some sample preparation is required for substances that exhibit high fluorescence (including some cutting agents). RS is ideal for both organic and inorganic species and can be used for both qualitative and quantitative analysis. Due to the similarity to IR (detecting forms of molecular movement to identify), Raman has similar issues with quantitative analysis. While quantitative analysis can absolutely be done with Raman spectroscopy, it can be a much more difficult process that may not be possible in a harm reduction setting. Due to the difficulty of quickly and easily performing quantitative analysis on many unknown samples, an important consideration for outreach is that portable handheld devices specifically designed to detect drugs of abuse are available. Qualitative results can be obtained in a fraction of seconds to several minutes.

The cost of a RS unit can vary widely (in the low thousands of dollars to US$50,000 and above). Like all of the previous devices, care must absolutely be taken in selecting the appropriate tool. Advanced knowledge is required for devices that are not optimized for drug testing.

#### How easy is it to use?

The level of expertise required to use this technology varies depending on the device, similar to IR. Some Raman spectrometers have been optimized for “point and shoot” action, giving a clear interpretation/reread of the substance(s) analyzed, and thus require merely basic to intermediate expertise for presumptive analysis. The requirements for quantitative analysis for portable “point and shoot” Raman spectrometers are similar to IR. Sorak et al. have also shown that some portable Raman spectrometers can offer quantitative analysis to a high degree of precision [25], although it must be stressed that this comes with the exact same considerations as the portable IR, as stated above. Other bench top or lab specific devices are most often not as simple and may require some database searching and interpretation of results. This can push the level of expertise required to intermediate, advanced, or expert, depending on the chosen device.

### X-ray diffractometry

#### How does it work?

In X-ray diffractometry (X-ray D), the drug sample is bombarded with high-energy X-ray radiation and crystalline atoms in the substance cause incident X-ray beams to diffract in various directions [30]. This allows for the determination of the spatial structure of molecules by measurement of how X-ray radiation is scattered by the molecular crystal lattice structure. By measuring the angles and intensities of the diffracted X-rays, it is possible to produce a three-dimensional picture of the density of electrons in the crystal, and, from this, it is possible to determine the positions of the atoms in the crystal as well as their chemical bonds and other structural information [30].

#### What substances can be detected and how accurately?

Any crystalline or partially crystalline substance (i.e., substances that are solid and usually either evidently crystalline or powder or pill, such as methamphetamine, ketamine, and cocaine) including those in mixtures and compounds with currently unidentified structure can be identified [31, 32]. This method is generally restricted to solid substances. X-ray D is used to identify precise chemical forms but not to quantify them. It can be used to identify diluents or adulterants [31]. This method is sensitive to both polymorphs and contaminants (common in illicit drugs). X-ray diffractometry determines structural information of the substance, so the substance can be identified with a very high degree of accuracy. This method is specific because substances have unique diffraction lines or an “X-ray fingerprint.” It is also sensitive in that drug concentrations and any additional agents used in cutting can be discerned through the obtained data. Studies have shown that this method can be used to identify a specific drug at only 5% of the total pharmaceutical formulation [33].

One benefit of X-ray D is that it requires no sample preparation and does not destroy the substance being tested. As well, only a very small sample size is needed (milligrams to micrograms) [31]. While it is the most reliable structural determination method and can determine the structure of currently unknown molecules, it is not suitable outside of a laboratory environment.

#### How easy is it to use?

X-rays are highly radioactive and very damaging to organic cells/DNA. Thus, this method requires a high level of training and safety procedures and is restricted to laboratory environments. The skill level involved in operation is advanced to expert.

## Least discriminatory

### Microcrystalline tests

#### How does it work?

These chemical tests result in the formation of unique microcrystals of a given analyte when a specific reagent is applied. The unique crystal formation is compared to a reference standard/control using a common light microscope. Microcrystals are compared based on shape, size, color, and spatial arrangement [34].

#### What substances can be detected and how accurately?

Several commonly abused substances can be identified, including cocaine, heroin, methadone, GHB (gamma hydroxybutyrate**)**, ketamine, phencyclidine, amphetamines, and methamphetamine [34]. With test reagents chosen to induce development of specific microcrystals with the analyte and a reference/control standard available, these tests can be highly specific as the crystals formed are a direct consequence of choice of reagent and analyte and are unique under these circumstances. This is provided that other substances do not react in a similar way, if at all, with the reagent, and provided that impurities, dilutents, and adulterants do not prevent or mask the formation of characteristic microcrystals for the drug tested. In these cases, a microcrystalline test can be considered highly characteristic but non-specific enough for a confirmatory test. Thus, this method is best suited to pure and/or separated samples. Sensitivity is high as samples require only micrograms of substance.

The benefit of microcrystalline tests is their relatively low cost. Minute amounts of reagents are required. Instrumentation is simple; however, this method does not quantify how much of a substance is present. Unfortunately, the sample that is tested is destroyed in the process, which may be less than ideal for people who are bringing the samples for identification.

#### How easy is it to use?

The expertise required is intermediate to advanced and requires adept interpretation of results.

### Thin-layer chromatography

#### How does it work?

Thin-layer chromatography (TLC) is a technique in which a sample is placed onto a planar stationary phase then a liquid mobile phase resulting in capillary action. The analyte is either adsorbed to the stationary phase or is in the mobile phase, and the time spent on the stationary phase or time spent in the mobile phase determines its retention time. Components of the sample travel at differing rates depending on the component’s size and affinity for the mobile phase [35]. The result is a plate of spots (separated components of the mixture) that have moved various distances on the stationary phase.

#### What substances can be detected and how accurately?

TLC can detect barbiturates, benzodiazepines, GHB, heroin, morphine, opium, oxycodone, and other opiates, amphetamines, cocaine, methamphetamine, MDMA (methylenedioxymethamphetamine or Ecstasy), ketamine, LSD, marijuana, mescaline, synthetic cannabinoids, and cathinones (commonly referred to as “bath salts”). Using TLC, it may be difficult to separate and identify novel psychoactive substances [36]. TLC performs fairly poorly at separating complex mixtures. Sensitivity is in the micro-nanogram range. Specificity can range from intermediate to high depending on the mixture, and measured retention factors can be used to make a preliminary identification of a substance but are not specific to a single compound [35]. In order to increase specificity in cases of similar retention factors, it must be used in conjunction with another technique such as Raman spectroscopy or colorimetric testing or in the case of UV active species, UV.

TLC is a relatively low-cost way to test substances and demonstrates good sensitivity and speed of separation. It can be used as a presumptive test with a fairly high degree of accuracy depending on sample purity. While TLC can identify some known substances in provided samples, it does not indicate (quantify) how much of a substance is present in the sample. TLC is best used in conjunction with a more discriminating technique such as Raman spectroscopy, MS, or IR.

#### How easy is it to use?

TLC is relatively simple to use and interpret and is thus suitable for basic to advanced skill level. This means that someone with basic skill may be able to perform a test following instructions but have trouble interpreting the results, whereas someone with intermediate to advanced skill level would have greater ability to interpret a test and could supervise basic skill level users.

### Spot/color tests

#### How does it work?

Spot/color tests offer presumptive testing based on chemical reactions between analytes and indicators. There are many possible indicator tests such as cobalt thiocyanate, Dille-Koppanyi, Duquenois-Levine, Mandelin, Marquis, nitric acid, para-dimethylaminobenzaldehyde, ferric chloride, Froehde, Mecke, Zwikker, and Simon’s (nitroprusside) [37]. The indicator chemically reacts with the analyte and causes a reaction that creates a certain color staining depending on the analyte tested. Spots are then compared visually with reference charts, the current standard being the Munsell color charts. There is a method that bypasses the human eye and its subjectivity by using a simple smartphone app to identify colors with high precision and accompanying software that matches the results in a searchable database [38]. This allows for a more precise quantitation of the color and therefore higher accuracy identification.

#### What substances can be detected, and how accurately?

Colorimetric tests exist for most drugs of abuse, including cocaine, various pharmaceutical opioids, amphetamines, LSD (lysergic acid diethylamide), cathinones (bath salts), heroin, and fentanyl. There may be other novel psychoactive substances that do not (yet) have any associated colorimetric tests. Each specific named test will have information on what analytes it can be used with. Unfortunately, the test also destroys the sample provided. That said, color tests do not require much sample: if it can be seen, it can be tested.

Colorimetric tests can be quite sensitive, with limits of detection in the microgram range depending on the spot test utilized and the analyte [37]. Multiple tests with multiple reagents can be used if a mixture of drugs is suspected, though each test requires in the low milligram range of substance and destroys the substance in testing. With the proper standards, these tests can be quite specific, although multiple analyses may be required for high specificity. Some knowledge about what the substance is supposed to be and about general appearance of certain substances can increase specificity. Colorimetric tests are considered presumptive, in that they can only identify presence or non-presence of a particular substance based on the test administered. A single test/reagent will only test for the presence or absence of a drug or class of drugs. A typical test is not sufficient for a suspected mixture or even an unsuspected mixture if there is any reason at all to have suspicion of the substance. An example battery test protocol for considerations of how to test a suspected mixture is included below.

Actual color results may vary depending on the concentration, whether the drug is in salt or free base form, additional diluents, or contaminants; positive result may indicate a specific drug or class of drugs present, but not always specific for a single drug or class. Colorimetric tests rely on simple chemical reactions and produce visible results that can be interpreted with the naked eye.

#### How easy is it to use?

Reagents and laboratory materials needed are inexpensive and readily available and can be performed with minimal training. Because each individual perceives color uniquely and because lighting conditions are not always optimal in non-lab settings, accuracy can be greatly enhanced with the use of smartphone apps to report color test results quantitatively [38]. Overall skill level required is basic to intermediate. A basic user can run the simple test and obtain results, whereas an intermediate user would run a standard protocol. An example of an intermediate protocol would be to run a battery of tests based on how much sample can be obtained without objection from the user. The tests should be based on an educated guess system, narrowing down possibilities through analysis and questions. Potential questions would be as follows: What did the user think it was or was told it was? What are recent novel substances that have been appearing in the clinic or on the street lately? What is the most dangerous substances worth testing for (smallest window of dosage)? Is there any knowledge of common mixtures, such as opioid mixtures?

The tests should be interpreted within a maximal 10-min window. The tests can be analyzed via smartphone or at least under good lighting if using the naked eye in order to most accurately determine color. The tests can then be matched against a database if a computer or the internet is available. From a system such as this, a presumptive test can then become a much more powerful tool.

### Immunoassay

#### How does it work?

Immunoassay involves the binding of an antibody that is selective for the drug or drug group of interest (antigen) and a label that will be part of the antibody-antigen complex that can be detected using some means (such as fluorescence). Antigen-antibody binding is based on a typical immune system response in which antibodies in biological tissue bind to antigens in order to neutralize or remove them. This technique is rarely used in drug analysis because these methods were originally designed for analysis in biological materials (primarily metabolites in urine). Thus, traditionally, immunoassay provides important patient information for clinicians but does not provide a determination of the type or amount of a drug prior to its ingestion/injection. ELISA can, however, be used to perform other types of biochemical assays in the detection of an analyte in a liquid sample. Very little scholarly information is easily accessible about which specific drugs ELISA can detect outside of biological samples (post ingestion/metabolization).

#### What substances can be detected and how accurately?

Various opioids and cocaine can be detected rapidly and somewhat effectively using immunoassay technology. There are problems with specificity regarding immunoassays, and there have been many instances of false positives due to similarity in drug structures or metabolites. Sensitivity is quite high with detection in the microgram range as antibody-antigen interactions occur on a molecular level [39].

#### How easy is it to use?

Immunoassay is fast and relatively inexpensive and in most instances, does not require high-level scientific knowledge to perform and interpret. Running such tests can require intermediate skill level. However, there is very little information available that has been scientifically published or available for public access on the usage of immunoassays for whole drug analysis. Immunoassay is most often employed to detect drug usage after the fact, such as in urine drug screens.

### Urine dipstick test

This method has recently come under attention as a relatively cheap, easy-to-use presumptive test for fentanyl [40]. A sample of the drug sample is dissolved in water, and if the drug contains fentanyl in a concentration above the cut-off levels, an indicator on the strip will appear. The methodology works via chromatographic immunoassay, and in the presence of an appropriate analyte, a strip on the indicator stick appears/changes color.

To date, fentanyl is the only drug for which this method of drug checking has been reported being used [25], and there is little published data about this methodology. There is no scientific data on sensitivity, although the strips have been developed to detect fentanyl in urine and are therefore specific to testing for fentanyl and/or fentanyl metabolites.

The provided sample is destroyed in the testing process. Urine dipsticks are very easy to use, quick to check, specific for fentanyl, proven in urine test situations, and recently been proven efficacious in testing unknown drug mixtures for the presence of fentanyl. However, dipsticks were designed for drug detection in urine, and therefore, due to low specific weight in other mediums, it may be possible that false positives occur.

Another potential concern with this method is that many retailers will only sell to health professionals, and thus, these items may be difficult to procure for harm reduction agencies unless they are affiliated with a health clinic. Some medical device companies may object to such a test being used in a harm reduction setting, even in the presence of qualified health professionals for liability reasons.

### Ultraviolet spectroscopy

#### How does it work?

This method is based on the absorption of light energy in the ultraviolet (UV) wavelength range. Light in this range can raise the energy levels of the electrons within a molecule from ground state to higher energy levels. Each transition to a higher energy level requires a given amount of energy, provided by light of a particular wavelength. Using a particular wavelength of light, a characteristic UV absorption spectrum can be obtained based on the electronic structure of the whole molecule as this structure will determine what wavelength(s) are absorbed versus which pass through a sample. UV-vis (ultraviolet visible) spectrophotometers measure the intensity of light passing through a sample and compare it to the intensity of light before it passes through the sample and capture this information to create a characteristic spectrum.

#### What substances can be detected and how accurately?

Drugs with similar structures may provide the same UV spectra. UV-vis has been used to identify MDMA, ketamine hydrochloride, cocaine hydrochloride, diazepam, phenobarbital, and barbital concentrations in the microgram range, as well as specifically identify six different compounds and for the first time, accurately discriminate some mixtures [41]. Other substances may be identifiable although literature is sparse on confirmatory usage for a broad spectrum of illegal drugs. UV spectrometry can be used on solid samples and therefore can be non-destructive in nature, although some samples may need preparation that can make them unsuitable for use afterwards. UV can be used quantitatively (amounts) and qualitatively (identification) and yields rough structural information providing modest selectivity to allow for some discriminating capability [24].

UV can be combined with chromatographic techniques for greater selectivity and specificity. It is not suitable for detection of several drugs in a mixture. Samples must be diluted or the technique can yield saturated spectra. Compounds lacking suitable chromophore provide no signal (for example, GHB has a low wavelength chromophore which makes analysis by UV-vis much more difficult without further sample preparation), although most drugs of abuse have a suitable chromophore due to aromatic ring structures in their chemical structures. Additionally, UV spectrum can vary depending upon the pH of the sample solution, and it is possible for chemical composition to change during the analysis. The level of expertise involved in UV is basic to advanced. The technique may be easily taught to someone with little to know theoretical knowledge of the technique, although interpretation of results would require intermediate to advanced knowledge.

## Conclusion

There are many variables to consider when selecting technology for drug checking on the front lines of harm reduction. Harm reduction agencies, if pursuing the addition of drug testing services, will need to consider not only the quantitative capabilities of the tests but whether the agency can afford the human and fiscal resources to support the use of the technology. Thus, the recommendations include a strong bias to cost-benefit and beg the important question of whether some of the less discriminatory interventions are better than no intervention at all. With these considerations in mind, the following recommendations will summarize the methods for drug testing at a point-of-care level.

The techniques that are the strongest candidates based on all considerations are IMS, IR, Raman spectroscopy, and spot/color tests, although these too have some associated drawbacks. Spot/color tests are purely presumptive. In most cases, quantitation is contingent on expert interpretation. In some cases, the therapeutic index is so small and such miniscule quantities can be used as an additive to mixtures that only the highest discriminatory techniques mentioned above are capable of proving unequivocally that the quantity present would fall in therapeutic index (i.e., would produce a high but not be fatal, barring extraneous circumstances).

In our review, the best methods for point-of-care drug testing are handheld IR or Raman spectroscopy. From a cost-to-benefit analysis, these methods (specifically the portable/handheld units) are superior in almost every way to every other method. Manufacturers have simply made these technologies extremely easy to use and effective at identification of unknown analytes. The major downsides of this technology are that quantitation may require advanced expertise and that these units are still fairly expensive. To use these units qualitatively usually requires very little technical expertise or training. Intended for use in the field, these units are small and portable and tend to be fairly rugged, while also being able to have near-lab identification ability [25]. While many of these devices are only currently in use in law/drug enforcement settings, use in harm reduction settings would be worth exploring.

IMS spectrometers are very robust and require minimal maintenance. They are routinely used in airports worldwide for narcotics detection. Training is easy and quick, and sensitivity and selectivity are very high. Consumables are cheap and have long lives. Sampling is non-destructive and quantification is possible without expert level understanding. Analysis is quick and accurate. IMS is the best option available for clinics with a moderate level of funding. Some gas analyzers allow online updating; rapid sample analysis of liquid, solid, and gas; and discrimination of multiple interfering species in a complex matrix. The capability to update online allows methodologies and new molecular species to be shared instantly among clinics enabling point-of-care testing to remain current.

Other methods worth considering for point-of-care drug testing are MS, TLC, and UV spectroscopy. MS is considered the current gold standard in forensic drug analysis. Since MS units have been in use for a long time, it is actually possible to obtain one for a decent price (low-to-mid thousands) in the used market. However, in order to obtain a newer device optimized for drug testing or for testing extremely low concentrations, it would come with a higher price tag, usually in the hundreds of thousands of dollars. This presents a difficulty of its own because of the wide range of machines available, it would take some considerable research at clinic level to determine the cost-benefit analysis of a new or used machine to ensure acquisition of a machine that is suitable for its intended purpose. Additionally, operation and maintenance of MS machines is still complex, so a clinic would have to assess training, operation, maintenance, and associated ongoing costs which may place such a device beyond the time and/or monetary costs to the clinic compared to the benefits provided.

UV spectroscopy and TLC are more affordable options, but also much less discriminatory. Both of these methods tend to be less technical in operation, maintenance, and interpretation of results, but also do not offer quantification at the same level of the more discriminatory methods. They are also less expensive than all of the more discriminatory techniques. However, when used in conjunction, TLC and UV can be quite powerful in identification of a wide variety of substances (including mixtures) and offer a more rudimentary quantification than the more discriminatory techniques.

A lower technology option is the spot/color tests, which are purely presumptive in nature, although they can be fairly specific at identification of a compound and/or mixture when utilizing a standardized procedure utilizing a battery of tests (as described above). Information about optimal technique can be easily accessed via the internet. Color tests are cost effective, fast to complete, and very easy to perform. The use of a smartphone app can aid in identifying the exact color profile. This can then be used in conjunction with a searchable database to perform the most accurate identifications. The fact that this technology is so cost-effective, easy to perform, and requires a very minute amount of substance makes it really stand out from many of the other presumptive methods [16]. This type of test is widely used in Europe [16]. These tests are not perfect and can be performed incorrectly. A proper standardization of technique should be implemented at the clinic level to maximize the accuracy of these tests.

Drug testing methods that are less suited to point-of-care drug testing situations include immunoassay, microcrystalline testing, and X-ray diffractometry. Immunoassays are traditionally designed for usage in biological samples as they work based on antibody-antigen interactions and as such are best suited for testing excreted metabolites (such as in urine). At best, an immunoassay can indicate the presence of drug(s), and at worst, they can give a high proportion of false positives. This may result in people using the substances anyways or serve to give the clinic a poor reputation, and users may soon stop going to the site for drug testing. That said, they are affordable and portable and can detect potentially fatal drugs like fentanyl.

Microcrystalline testing is a highly limited method as the drug needs to be mostly (or completely) pure. This testing has no quantification capabilities at all and requires high skills and knowledge to identify drugs based purely on crystal structure. X-ray diffractometry is a highly discriminating testing method; however, this method basically requires partnership with a specialized lab/institution. X-ray diffractometers are incredibly expensive (mid-to-high tens of thousands), difficult to maintain and operate, and have the added factor of using radioactivity which may present health and safety concerns.

There is a wide variety of techniques that have been validated for drug identification and/or quantification. Each of these techniques has a variety of associated pros and cons that must be considered. With this in mind, this review is not meant to be an in-depth rigorous scientific treatment of each of these methods, but a guide for the practical consideration of usage and recommendations for point-of-care harm reduction purposes. It is sincerely expected that this document will help to narrow down consideration of each of these techniques and that each clinic would then determine a smaller subset of techniques to consider implementing. It would be prudent for clinics that can obtain the funding to contact the companies who produce and design these devices and discuss possible usage in a harm reduction setting as many of the devices are only currently in use in law enforcement and research.

## Abbreviations

API: Active pharmaceutical ingredients
FAIMS: Field asymmetric ion mobility spectrometer
GC: Gas chromatography
GHB: Gamma hydroxybutyrate
IMS: Ion mobility spectrometry
IR: Infrared
LC: Liquid chromatography
LSD: Lysergic acid diethylamide
MDMA: Methylenedioxymethamphetamine or Ecstasy
MS: Mass spectrometry
RS: Raman spectroscopy
SWGDRUG: Scientific Working Group for the Analysis of Seized Drugs
TLC: Thin-layer chromatography
UV: Ultraviolet
UV-vis: Ultraviolet visible
X-ray D: X-ray diffractometry
